# Microglia in Alzheimer’s Disease: From Homeostatic Guardians to Multifaceted Drivers of Neuropathology

**DOI:** 10.3390/cells15141291

**Published:** 2026-07-18

**Authors:** Lizhen Ma, Yan Zhao, Chaochan Cai, Qing Liu, Xiangjun Hu, Lifeng Wang

**Affiliations:** 1 Academy of Millitary Medical Sciences, Beijing 100850, China; malizhen_2022@163.com (L.M.); qingliu670214@163.com (Q.L.); 2 Beijing University of Chinese Medicine, 11 North Third Ring East Road, Beijing 100029, China; 20240931355@bucm.edu.cn (Y.Z.); 202453036@bucm.edu.cn (C.C.); 3 School of Life Science and Food Engineering, Hebei University of Engineering, Handan 056038, China

**Keywords:** AD, microglia, neuroinflammation, metabolic reprogramming, TREM2, disease-associated microglia, glial interactions, precision immunotherapy

## Abstract

**Highlights:**

**What are the main findings?**

- Microglia exhibit dual functional states that transition dynamically during AD progression, shifting from homeostatic guardians to pathological drivers.
- Multiple signaling pathways coordinate microglial Aβ and tau clearance versus propagation.

**What are the implications of the main findings?**

- Precise targeting of microglial functional states offers a novel disease-modifying strategy for AD.
- Modulating glial–glial interactions and reprogramming microglial metabolism hold promise for AD prevention and treatment.

**Abstract:**

**Background:** Alzheimer’s disease (AD) affects >55 million people worldwide and lacks disease-modifying therapies. Microglia, the CNS resident immune cells, dynamically transition between protective and pathological states during AD progression. Recent advances in single-cell sequencing and metabolomics reveal that microglial roles extend beyond simple M1/M2 polarization. This review synthesizes these advances into a framework integrating microglial plasticity, metabolic reprogramming, and intercellular communication in AD. **Methods:** We reviewed recent (2020–2026) studies on microglial biology in AD, focusing on DAM (disease-associated microglia) ontogeny, metabolic reprogramming, immune checkpoints, and glial crosstalk. **Results:** Microglia exhibit spatiotemporal heterogeneity, shifting from protective phagocytic phenotypes (M2, DAM1/2) in early AD to pro-inflammatory and exhausted states (M1, terminal inflammatory microglia [TIM], lipid droplet-accumulating microglia [LDAM]) as pathology advances. Key pathways—TREM2/SYK phagocytosis, Piezo1 mechanotransduction, TAM (Tyro3, Axl, Mer) receptor signaling, and metabolic regulators (HK2, iron, APOE4-driven lipid metabolism)—orchestrate these transitions. Microglia also interact with astrocytes, T cells, and peripheral immune cells via IL-3, complement C3, MHC-I and MHC-II, forming glial-immune networks that modulate Aβ clearance, tau propagation, and synaptic integrity. **Conclusions:** Precisely targeting microglial functional states, rather than broad immunosuppression, is a promising disease-modifying strategy for AD. Future therapies should integrate metabolic reprogramming, glial network regulation, and immune checkpoint modulation to preserve protective microglial phenotypes in early AD while suppressing pathological activation and exhaustion in advanced disease.

## 1. Introduction

AD is the leading cause of dementia worldwide, affecting over 55 million individuals and imposing annual costs exceeding $1 trillion globally. As populations age, these figures are projected to triple by 2050, underscoring the urgent need for disease-modifying therapies [1]. Despite decades of research focused primarily on amyloid-β (Aβ) and tau pathologies, effective treatments remain elusive, necessitating a paradigm shift toward understanding the brain’s innate immune system as a central driver of neurodegeneration [2].

**Figure 1:**
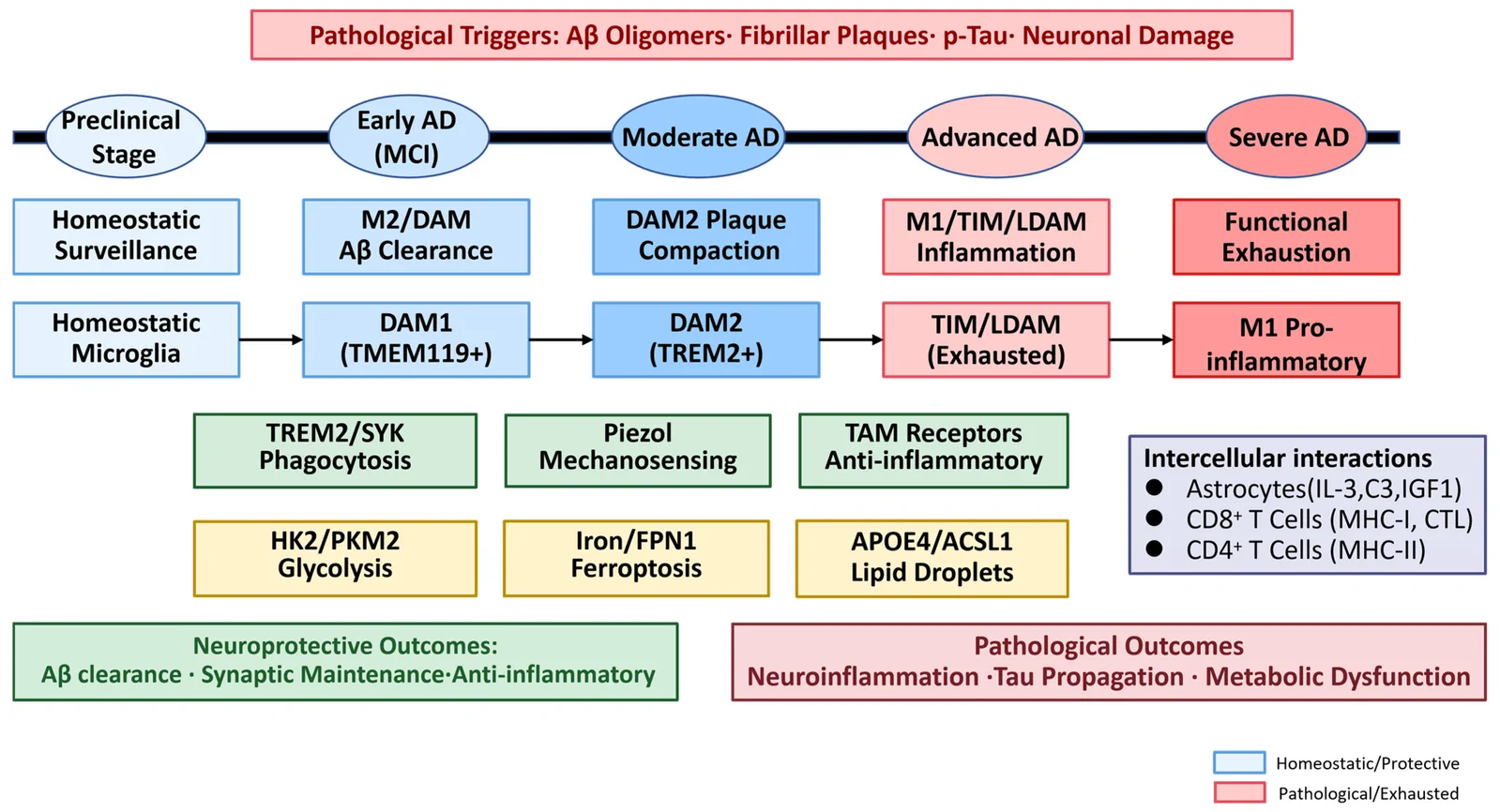
Microglial Functional States and Key Regulatory Networks in AD. This schematic illustrates the spatiotemporal transition of microglial phenotypes across AD progression, from early homeostatic and M2/DAM states to late-stage M1, TIM, and LDAM phenotypes. Key molecular pathways (TREM2/SYK, Piezo1, TAM receptors, metabolic reprogramming, and glial–glial crosstalk) that orchestrate these transitions are annotated, along with emerging therapeutic intervention points (e.g., PTP1B inhibitors, Piezo1-targeted nanobubbles, TIM-3 modulators, and iron chelators). Precise stage-dependent modulation of microglial states, rather than uniform suppression or activation, represents the optimal therapeutic strategy.

Microglia, the brain’s resident macrophages, constitute the primary immune effectors of the central nervous system. Beyond their traditional role in pathogen defense, they actively participate in synaptic pruning, metabolic regulation, debris clearance, and intercellular communication with astrocytes, neurons, and peripheral immune cells [3]. In AD, microglia exhibit remarkable functional plasticity: they can either restrain pathology through phagocytosis and anti-inflammatory signaling or accelerate neurodegeneration via chronic inflammation and metabolic dysfunction [4]. Recent single-cell and single-nucleus RNA sequencing studies have revealed unprecedented heterogeneity among microglial subpopulations, challenging the classical M1/M2 dichotomy and identifying novel states such as disease-associated microglia (DAM), terminal inflammatory microglia (TIM), and lipid droplet-accumulating microglia (LDAM) [5]. A schematic overview of these functional states and their key regulatory networks is presented in Figure 1.

While previous reviews have largely focused on single aspect of microglial biology—such as neuroinflammation or phagocytosis—this paper provides an integrated, multi-dimensional framework. We systematically elucidate microglial roles across four interconnected aspects: (1) neuroinflammatory regulation and immune crosstalk; (2) dual mediation of Aβ clearance and plaque formation; (3) metabolic homeostasis maintenance (iron, glucose, and lipid metabolism); and (4) glial network interactions. By synthesizing recent advances in microglial mechanobiology, metabolic reprogramming, and intercellular communication, we identify novel therapeutic windows and propose that precision modulation of microglial functional states—rather than broad activation or suppression—represents the most promising path forward for AD immunotherapy.

## 2. Methods

A comprehensive literature search was performed in the PubMed databases for articles published between January 2020 and June 2026. The search strategy employed a combination of the following keywords: (“microglia” OR “microglial”) AND (“Alzheimer’s disease” OR “AD” OR “Alzheimer”) AND (“disease-associated microglia” OR “DAM” OR “TREM2” OR “phagocytosis” OR “neuroinflammation” OR “metabolic reprogramming” OR “glial interactions” OR “tau pathology” OR “amyloid-β”). Additional relevant studies were identified by manual screening of the reference lists of included articles and key reviews. Studies were eligible for inclusion if they were: (1) original research articles, clinical trials, or mechanistic reviews that directly addressed microglial biology in the context of Alzheimer’s disease; (2) published in English; and (3) provided sufficient methodological detail to allow critical appraisal of the findings. Conference abstracts, case reports, editorials, and studies not primarily focused on microglia or AD pathology were excluded.

## 3. Neuroprotection and Inflammatory Response in Microglia

As the primary immune cells of the central nervous system, microglia exhibit dynamic functional transitions during the pathological progression of AD. Microglia, as the primary immune cells of the central nervous system, exhibit dynamic functional transitions during the pathological progression of AD. Traditionally, they are broadly categorized into M1-type (classically activated, pro-inflammatory) and M2-type (alternatively activated, anti-inflammatory) based on their activation phenotypes [6]. M1 microglia predominantly release pro-inflammatory cytokines such TNF-α and IL-6, exacerbating neuroinflammation and inducing neuronal damage [7]. Conversely, M2 microglia secrete anti-inflammatory mediators and neurotrophic factors, exerting neuroprotective effects [8]. In the early stages of AD, M2-type microglia predominate, maintaining neuronal microenvironmental stability by clearing Aβ and abnormally phosphorylated tau protein [9]. As the disease progresses to advanced stages, microglia progressively shift towards the M1 phenotype, enhancing pro-inflammatory factor release and exacerbating neuronal damage, thereby driving the pathological progression of AD [10]. Notably, while the M1/M2 dichotomy provides an initial framework for understanding microglial activation mechanisms, recent advances in single-cell sequencing have revealed high heterogeneity and functional continuity among microglia throughout disease progression. Studies indicate that the evolutionary trajectory of microglial states extends far beyond simple polarized classifications, manifesting instead as a more complex continuum of functional lineages.

In AD, the aggregation of Aβ and tau proteins, along with the damage induced by these aggregates, sequentially triggers activation of the brain’s parenchymal immune system. This involves alterations in astrocytes, microglia, brain-boundary immune cells, and meningeal immune cells, alongside dysfunction of the lymphatic drainage system. Dysfunction of these protective barriers within the brain may lead to the ectopic migration of peripheral immune cells—particularly T cells—into the parenchyma, thereby establishing a novel immune environment within the AD brain [11]. Mehdi Jorfi et al. utilized a constructed human three-dimensional neuroimmunological axis model to discover that T cells and monocytes selectively infiltrate AD cultures. The infiltration of CD8^+^ T cells can lead to microglial activation, exacerbate neuroinflammatory responses, and promote neurodegeneration, thereby accelerating the pathological progression of AD [12]. Chen et al. observed a marked increase in both microglia, CD4^+^ T cells and CD8^+^ T cells within the brains of PS19-AD mice, predominantly concentrated in regions exhibiting tau hyperphosphorylation and neuronal loss (the hippocampus and piriform cortex). Close communication exists between microglia and T cells within the AD brain. Microglia not only function as innate immune cells but also serve as crucial antigen-presenting cells within the brain, participating in the brain’s cellular immunity [13]. The interaction between microglia and T cells (CD4^+^ T cells and CD8^+^ T) within the AD brain activates adaptive immune responses (humoral and cellular immunity), constituting a core driver of neuronal death caused by neurofibrillary tangles. Recent studies reveal that microglia within the AD brain can induce the infiltration of peripheral CD4^+^ T cells and CD8^+^ T cells into the brain via chemokines (CCL2, CCL3, CCL4, CXCL10, etc.), with a particularly pronounced effect on CD8^+^ T cell recruitment [14]. Microglia may interact with CD8^+^ T cells via MHC class I molecules, thereby activating CTL (Cytotoxic T Lymphocyte) immune responses and exacerbating AD pathological manifestations, and interact with CD4^+^ T cells via MHC class II molecules, activating relevant humoral and cellular immunity. Conversely, downregulating MHC class I and MHC class II molecule expression alleviates tau-related pathological alterations.

Microglia serve as multifaceted regulators of AD pathogenesis, with their functional impact shifting dynamically across disease stages and anatomical contexts. In the neuroinflammatory domain, microglia transition from homeostatic surveillance to M1-type pro-inflammatory activation, driven by Aβ and tau pathology as well as peripheral CD4^+^ T cell and CD8^+^ T cell infiltration. This establishes a self-perpetuating inflammatory cascade involving NLRP3 inflammasome activation, complement signaling, and MHC-I-mediated cytotoxic T lymphocyte responses that collectively drive neuronal death.

## 4. Microglia Mediate Dual Effects of Aβ Clearance and Plaque Formation in AD

Microglia also play a dual role in regulating Aβ metabolism in AD. In their resting state, microglia participate in immune surveillance within the central nervous system; upon activation, their functional state determines the direction of their influence on Aβ metabolism. Moderately activated microglia promote Aβ clearance and exert neuroprotective effects; conversely, abnormal or sustained activation may induce chronic neuroinflammation, exacerbating neuronal injury and synaptic loss, thereby driving disease progression [15].

### 4.1. Phagocytosis and Barrier Function of Aβ by Microglia

As the resident phagocytes of the central nervous system, microglia can be activated into DAM, regulating the structure and toxicity of Aβ plaques through a triggering receptor expressed on myeloid cells 2 (TREM2)-dependent mechanism [16]. These cells establish a physical barrier between Aβ plaques and the surrounding neurofibrillary tangles, thus curtailing the propagation of plaques and ameliorating their deleterious effects on adjacent neurons [17]. Furthermore, microglia enhance their phagocytic capacity for Aβ by expressing scavenger receptors (such as SR-A, SR-B) and molecules like TREM2, thereby reducing its deposition within the brain parenchyma [18]. TREM2 deficiency impairs the conversion of microglia to the DAM phenotype, diminishing their coverage of Aβ plaques. This leads to increased accumulation of more toxic fibrillar plaques, exacerbating dendritic atrophy [19].

### 4.2. Key Pathways for Microglial Regulation of Aβ Metabolism

The process by which microglia are activated into DAM under the pathological stimuli of AD exhibits a distinct two-stage character: The initial phase (DAM1) is TREM2-independent, whereas subsequent transition to the effector phase (DAM2) is highly dependent on signaling via the triggering receptor expressed on TREM2 [20]. TREM2 plays a central role in microglia-mediated regulation of Aβ metabolism. Firstly, TREM2-dependent DAM forms a physical barrier between Aβ plaques and the surrounding neural network, limiting plaque spread and mitigating their toxic effects on neighboring neurons [17]. Secondly, TREM2 activates downstream signaling pathways by binding to Aβ and its associated ligands (such as APOE), thereby enhancing phagocytic activity in microglia and promoting Aβ clearance [21]. Research indicates that TREM2 may also act synergistically with other phagocytosis-related receptors (such as scavenger receptors SR-A and SR-B) to collectively reduce Aβ deposition within the brain parenchyma [18]. TREM2 loss-of-function severely impairs the conversion of microglia to the DAM phenotype, diminishing their encapsulation of Aβ plaques. This leads to increased accumulation of more toxic fibrillar plaques and exacerbates neurotrophic degeneration [19]. Notably, TREM2 not only plays a role in Aβ pathology but also exerts significant functions in regulating tau pathology. Recent studies indicate that overexpression of human wild-type TREM2 moderately reduces phosphorylated tau levels and partially preserves neuronal integrity, whereas TREM2 carrying the R47H risk variant exhibits a loss-of-function phenotype with no significant impact on tau pathology or microglial activation [22]. Beyond TREM2, the functional role of the microglia-specific marker TMEM119 in AD pathogenesis has recently been elucidated. Studies demonstrate that TMEM119 directly binds to Aβ oligomers, forming a ternary complex with the Aβ clearance receptor LRP1 to promote Aβ degradation via the lysosomal pathway. Functionally, TMEM119 primarily maintains microglial homeostasis and regulates their transition to the DAM1 stage, whereas TREM2 governs the shift from DAM1 to the phagocytic DAM2 stage [23]. This discovery offers new insights into the molecular mechanisms governing microglial fate regulation.

Spleen tyrosine kinase (SYK) signaling plays a pivotal role in regulating Aβ metabolism within microglia. Studies indicate that SYK signaling deficiency leads to increased Aβ protein accumulation in brain regions such as the cortex, hippocampus, and thalamus. It simultaneously impairs the efficiency of microglia in compacting Aβ into dense plaques, resulting in Aβ plaques exhibiting more diffuse and less dense structural characteristics, thereby accelerating disease progression in AD model mice [24]. Recent research has further elucidated the regulatory mechanisms of the SYK signaling pathway and its therapeutic potential. Recent studies have discovered that protein tyrosine phosphatase 1B (PTP1B), acting as a key negative regulator of SYK, inhibits phagocytic function in microglia by directly dephosphorylating SYK. In APP/PS1 mice, either knocking out the PTP1B gene or employing its allosteric inhibitor DPM-1003 enhances SYK phosphorylation levels. This promotes the transition of microglia towards an immunologically activated and phagocytic phenotype, improves energy metabolism adaptability, thereby reducing Aβ burden and enhancing cognitive function [25]. Moreover, newly identified compounds through drug screening—new chlorogenic acid and L-arabinitol—can induce the conversion of microglia towards an M2 protective phenotype by upregulating SYK gene expression. This enhances their chemotaxis and phagocytic capacity towards Aβ, whereas silencing SYK expression significantly attenuates these effects [26]. In the context of tauopathies, Clec7a, a microglial marker associated with the disease, has been shown to modulate synaptic loss by activating SYK signaling. In a study of PS19 tauopathy mice, the progressive activation of the Clec7a-SYK signaling axis was found to correlate with disease progression. However, Clec7a blockade was observed to mitigate microglial hyperactivation, suppress neuroinflammation, and restore synaptic homeostasis [27]. It is noteworthy that the newly developed chimeric antigen receptor microglia (CAR-M) therapy has also demonstrated that activation of the Clec7a-SYK signaling pathway significantly enhances the degradation capacity of intracellular Aβ oligomers [28]. Collectively, these studies reveal the pivotal role of SYK as a core regulator of microglial function. The targeting of the PTP1B-SYK signaling axis, or the direct activation of SYK, has emerged as a novel immunotherapeutic strategy for AD, promoting Aβ clearance and improving cognitive function. The mechanosensitive ion channel Piezo1, which is considered a key molecule for microglia to perceive the mechanical properties of the microenvironment, also participates in regulating the phagocytic activity of microglia. Piezo1 deficiency has been demonstrated to diminish microglial uptake and clearance of Aβ fibrils, resulting in loosened plaque architecture and promoting outward extension of fibrillar structures surrounding plaques [29]. This suggests that mechanical signal transduction plays a critical role in microglial Aβ clearance. The stiffness of Aβ fibrils can be sensed by the Piezo1 channel in microglia, thereby activating downstream calcium signaling to regulate both the aggregation of microglia towards Aβ plaques and their phagocytic function [30]. In vitro models of Aβ plaque-associated microenvironments have been demonstrated to illustrate that Aβ stiffness instigates early activation of microglia via the PIEZO1 mechanotransduction pathway, thereby engendering an oxidative stress microenvironment that subsequently compromises microglial membrane integrity [31]. Therapeutic interventions that target Piezo1 are emerging as a leading research direction in the field of AD. The research team developed magnetic nano-bubbles (PT-MNBs) that targeted Piezo1 in microglia. The transcranial magneto-acoustic coupling stimulation technique involves the non-invasive focalisation of physical energy into deep brain regions, thereby inducing the PT-MNBs to generate responsive mechanical and electrical stimulation signals. This strategy achieves glial cell-specific anchoring via surface-modified phosphatidylserine, whilst the conjugated anti-Piezo1 antibody precisely delivers the electromechanical stimulus to Piezo1 channels within microglia. This results in the synchronous activation of both the mechanosensitive and voltage-sensitive domains of Piezo1, leading to the recruitment of microglia to inflammatory sites and the enhancement of Aβ clearance [32].

TAM (Tyro3, Axl, Mer) family receptors have been identified as significant molecules in the recognition and phagocytosis of Aβ plaques by glial cells, thereby playing a crucial role in the regulation of AD pathology [33]. Recent studies have further elucidated TAM receptor-mediated neuroprotective mechanisms: Tyro3 overexpression significantly suppresses pro-inflammatory cytokine IL-1β production in both the AD microenvironment (tau+Aβ42) and classical NLRP3 inflammasome models by enhancing STAT1 phosphorylation and downregulating the IL-1β transcription enhancer C-EBP-β. In AD mouse models, the activation of TAM signaling via Gas6-α-Aβ lentiviral injection has been shown to result in a significant reduction in IL-1β levels in the brain. This suggests that TAM receptors may have both anti-inflammatory functions and a role in regulating Aβ phagocytosis [34]. Research indicates that the TAM receptor and its ligand serve as the primary mediators for microglia to recognize newly formed ‘loose’ Aβ deposits [35]. The phagocytic process they mediate has been shown to reduce AD pathology by trimming and compacting plaques, thereby limiting the subsequent spread of tau pathology. It is noteworthy that this phagocytic process itself may drive the formation of high-density “dense-core” plaques [35]. Relevant studies indicate that inhibiting microglial function significantly reduces the formation of Aβ deposits in the brains of AD model mice, with residual “dense-core” plaques appearing only in regions where microglial aggregation persists [36]. It is therefore hypothesized that microglial phagocytosis of Aβ and subsequent intracellular processing (such as Aβ aggregation induced in the acidic lysosomal environment) may promote the formation of these dense-core plaques. These plaques may be expelled via exocytosis or released extracellularly following microglial apoptosis [37]. From a pathophysiological perspective, the formation of these ‘dense core’ plaques may represent a compensatory protective mechanism against chronic inflammation and neurodegenerative changes. In recent years, TAM receptors have garnered significant attention as emerging targets for glial cell-specific therapeutics [38]. Research has revealed that elevated levels of soluble TAM receptors (Tyro3 and Axl) in the cerebrospinal fluid of AD patients correlate with cognitive protection [34], suggesting that enhancing TAM signaling may represent an effective strategy for ameliorating neuroinflammation in AD. Current therapeutic approaches targeting TAM receptors primarily encompass: activating TAM signaling via Gas6 fusion proteins, enhancing receptor function through gene overexpression, and synergistically promoting Aβ phagocytosis by microglia in combination with anti-Aβ antibodies [39]. These studies establish a theoretical foundation for developing novel TAM receptor-based immunotherapeutic strategies for AD.

### 4.3. Novel Phenotype of Microglial Functional Exhaustion: Terminal Inflammatory Microglia

Recent studies have revealed a novel phenotype of functional exhaustion in microglia within the pathological progression of AD. The research team led by Sohail Tavazoie at Rockefeller University discovered a subpopulation of functionally exhausted TIM, in the brains of aged mice and mice carrying the APOE4 gene variant. This phenomenon has also been validated in human AD patient datasets [40,41]. Furthermore, TIM cells have been observed to lose their capacity to effectively clear Aβ plaques, and to show significant enrichment in APOE4-carrier mice. This suggests a close association with AD-related immune dysfunction [40,41]. Recent single-cell sequencing studies have further elucidated the molecular characteristics of TIM cells and their dynamic changes during the progression of AD. Research findings have indicated that TIM-3 (encoded by the HAVCR2 gene), functioning as an immune checkpoint molecule, exhibits aberrant expression in AD microglia. This study delineates a TIM-3-positive microglial subpopulation that exhibits dual pathogenic mechanisms. Firstly, it drives persistent neuroinflammation via TNF-α/NF-κB and IL-6/JAK/STAT3 signaling pathways. Secondly, it impairs Aβ phagocytic function through PI3K-AKT and FcγR pathways [42]. Based on the hypothesis that immune exhaustion leads to plaque accumulation, restoring the functional state of TIM cells may offer novel therapeutic strategies for AD. In-depth exploration of key signaling molecules regulating TIM formation (such as TIM-3 and its downstream pathways), coupled with the development of targeted therapeutics (e.g., ATRA or specific TIM-3 modulators) to intervene in this process, holds promise for maintaining microglial homeostasis and thereby delaying cognitive decline [43]. Recent studies indicate that synthesizing SER-modified microglia can simultaneously clear Aβ and suppress inflammatory responses, offering a novel technical pathway for reversing microglial functional exhaustion [44]. The major molecular pathways governing microglial Aβ clearance and plaque formation, including their primary functions, effects on Aβ pathology, and emerging therapeutic targets are shown in Table 1.

## 5. The Role of Microglia in the Pathological Spread of Tau Protein

The role of microglia in the propagation of tau pathology is both complex and pivotal. By engulfing, processing, and releasing biologically active tau “seeds”, they profoundly influence the dissemination of pathological tau within the brain and the progression of disease. Tau has been demonstrated to mediate inflammatory responses in neurodegenerative diseases and to activate microglia [45,46].

### 5.1. Molecular Mechanisms Underlying Microglia-Mediated Spread of Tau Pathology

Research indicates that aggregated tau protein can activate NLRP3-ASC inflammasomes in a manner analogous to prion protein seeding. This, in turn, induces microglial activation and the release of pro-inflammatory cytokines, leading to damage to the intracellular environment. Concurrently, activated microglia may further exacerbate the spread of tau pathology by disrupting dendritic and axonal structures [46]. Recent research has revealed the pivotal role of Galectin-9 (Gal-9) in this process: tau fibrils can induce increased expression and secretion of Gal-9 in microglia. The secreted Gal-9 has been shown to act as a ligand for Toll-like receptor 4 (TLR4), thereby activating the NLRP3 inflammasome and promoting the release of neurotoxic cytokines such as IL-1β and IL-18 [47]. In the context of tau P301S transgenic mice, the Gal-9 gene knockout has been shown to significantly attenuate microglial activation and tau deposition, while concurrently protecting synaptic integrity and enhancing cognitive function [48]. Conversely, the intracerebral injection of Gal-9 has been observed to exacerbate tau pathology. These findings reveal the pivotal role of the ‘tau-Gal-9-TLR4-NLRP3’ positive feedback loop in driving tau pathological propagation. Microglia can directly or indirectly modulate tau phosphorylation levels and/or the propagation of pathological tau through multiple surface receptors, intracellular inflammatory signaling pathways, and the release of exosomes or pro-inflammatory factors [49,50]. Recent studies have further elucidated the specific mechanisms by which microglia clear tau protein. Research using microglia derived from human induced pluripotent stem cells revealed that monomeric and fibrillar tau are internalized via distinct cellular mechanisms and exhibit different clearance kinetics: fibrillar tau persists intracellularly significantly longer than monomeric tau. It is important to note that while acute inflammatory activation alters total tau endocytosis, it does not affect the rate of intracellular tau clearance. This finding indicates that the endocytic-lysosomal system, rather than the inflammatory polarization state, is the primary bottleneck for fibrillar tau clearance [51]. Table 2 delineates the diverse microglial states implicated in tau pathology propagation and clearance, highlighting their distinct molecular markers, functional mechanisms, and therapeutic implications.

### 5.2. The Dual Role and Functional Exhaustion of Microglia

It is noteworthy that microglia exhibit a dual role in responding to tau protein. In the event of failure to adequately degrade internalized tau into non-toxic forms, it is possible that tau seeds might be released inside the body, thus facilitating their transmission between neighboring cells. Consequently, under certain conditions, microglia may paradoxically become drivers of tau pathology propagation. Recent research in human-mouse chimeric AD models has revealed that combined Aβ and tau pathology can induce human-derived microglia to produce robust type I interferon and pro-inflammatory cytokine responses [52]. This further promotes the formation of a distinct ‘rod-like’ microglial phenotype, which is closely associated with tau pathology and neurodegeneration.

### 5.3. Therapeutic Strategies Targeting Microglia

Recent studies have indicated that long-term NRF2-driven microglial repopulation has the potential to mitigate microglial proliferation, neuronal loss, and cognitive deficits in mice models of tauopathies. Following microglial depletion via colony-stimulating factor 1 receptor (CSF1R) inhibitors, combined repopulation with NRF2 activation has been shown to preserve cognitive function, prevent hippocampal neuronal loss, and restore microglial phenotype.

Transcriptomic analysis further reveals that this combined therapy modulates tau-associated mitochondrial gene expression alterations. Furthermore, therapeutic studies of the immunomodulator GV1001 in PS19 tau transgenic mice are underway, with microglial activity and treatment response being monitored via markers including TSPO-PET, tau-PET, and cerebrospinal fluid sTREM2 [53]. This finding offers the potential for the provision of new evidence-based support for immunomodulatory therapies. It is evident that microglia play a multifaceted role in the propagation and regulation of tau pathology, utilizing a variety of mechanisms to do so. It is imperative to obtain a comprehensive understanding of the functional state transitions that occur across various disease stages, as well as the molecular regulatory networks that govern them. This understanding promises to unveil novel therapeutic approaches for intervening in tau pathology by precisely modulating microglial immune functions. It is hypothesized that enhancing microglial phagocytic and clearance capacities, modulating the expression of relevant surface receptors, and controlling the release of inflammatory cytokines may effectively reduce the accumulation and propagation of pathological tau proteins. This approach has the potential to delay the pathological progression of AD and alleviate associated symptoms, holding significant promise for developing AD-modifying therapeutic strategies targeting microglia.

Regarding proteinopathy, microglia exert context-dependent dual effects. TREM2-dependent DAM formation, SYK-mediated phagocytosis, and TAM receptor signaling facilitate Aβ clearance and plaque compaction. However, functional exhaustion (TIM), iron overload, and exosomal release of tau seeds transform microglia into drivers of pathology propagation.

## 6. Role of Microglia in AD Brain Metabolism

Metabolic dysregulation constitutes one of the key pathological hallmarks of AD, involving multiple aspects such as iron homeostasis imbalance, energy metabolism reprogramming, and lipid metabolism abnormalities. Microglia play a central role in cerebral iron metabolism. As the cell type most adept at accumulating and storing iron within the central nervous system [54], microglia maintain iron homeostasis by expressing multiple iron transporters and metabolic proteins, including transferrin receptors, divalent metal transporter 1 (DMT1)and ferritin [55,56]. Compared to neurons, microglia possess a more efficient iron accumulation capacity, making them a key hub for regulating iron metabolism within the brain.

### 6.1. Bidirectional Regulatory Mechanism Between Microglia and Iron Metabolism

Iron metabolism disorders are a common feature across a range of neurodegenerative diseases, including AD [57]. Recent studies have further revealed that iron homeostasis imbalance may exacerbate neurodegenerative pathological processes by promoting ferroptosis [58,59]. Iron accumulation is observed in multiple brain regions of AD patients, with markedly elevated iron concentrations within senile plaques, suggesting that cerebral iron accumulation constitutes an early pathological hallmark of AD [60]. Recent studies demonstrate that iron deposition in the brain exhibits regional specificity and correlates closely with AD pathological burden: for instance, ferritin levels in the amygdala explain 66% of TDP-43 pathological variation in AD patients, while ferritin levels in the hippocampus effectively distinguish AD patients from healthy controls [61]. Increased blood–brain barrier permeability and neuroinflammation are key drivers of cerebral iron accumulation [62]. Furthermore, blood–brain barrier disruption and chronic inflammation mutually reinforce each other, further disrupting iron homeostasis and jointly propelling AD pathological progression.

A complex bidirectional regulatory relationship exists between microglia and iron metabolism. On the one hand, excessive microglial activation produces substantial neuroinflammatory cytokines, thereby promoting iron uptake and activation. Subsequent downregulation of iron-binding proteins leads to intracellular iron sequestration [63]. Dysfunction in microglia has been demonstrated to disrupt iron metabolism, thereby amplifying iron-induced neuronal degenerative changes [64]. Conversely, elevated iron levels within the brain also influence the phenotype and function of microglia. The activated state of microglia may in turn affect iron metabolism, creating a vicious cycle that exacerbates neurodegenerative processes in AD [65].

Iron accumulation pushes microglia toward an inflammatory and glycolytic phenotype, and this glycolytic phenotype is associated with compromised phagocytic ability [66]. In AD models, overexpression of heme oxygenase-1 (HO-1) in microglia leads to iron toxicity accumulation, elevates reactive oxygen species (ROS) levels, and reduces glutathione peroxidase 4 (GPX4) expression, ultimately inducing ferroptosis and memory impairment [67]. Recent studies further elucidate molecular networks regulating microglial iron metabolism: activation of TRPM8 channels enhances transferrin-mediated iron uptake, leading to mitochondrial iron overload and ferroptosis, suggesting its potential as an intervention target [68]; M2 microglial exosomes activate Wnt/β-catenin signaling via TREM2, upregulating GPX4 and FTH1 expression and thereby alleviating neuronal ferroptosis and neuroinflammation [69].

Iron overload has also been demonstrated to induce microglial senescence, as evidenced by elevated expression of cell cycle arrest markers (P21, P16), increased senescence-associated β-galactosidase activity, and M1-type polarisation [54]. 27-Hydroxycholesterol, the predominant oxycholesterol in peripheral blood, accumulates in the brain via the blood–brain barrier. The disruption of iron homeostasis is achieved through the modulation of the DMT1/ferritin/GPX4 axis, with the resultant elevation of ROS levels and impairment of mitochondrial function. thereby promoting microglial senescence and neuroinflammation [70]. The iron chelator deferoxamine mitigates microglial senescence and ferroptosis, suggesting that regulating iron metabolism may represent a novel strategy for improving cognitive function in AD. Furthermore, recent studies demonstrate that carbon ion beam stimulation (CIS) therapy reduces iron species in AD mouse brains by 33–60% in a dose-dependent manner, concurrently downregulating neuroinflammation, upregulating anti-inflammatory responses, improving memory, and enhancing hippocampal neurogenesis [71]. Collectively, these findings establish the central role of glial iron metabolism dysregulation in AD pathology. Targeting iron homeostasis regulation (via chelators, GPX4 activators, TRPM8 modulators, or CIS physical intervention) and enhancing M2-type exosomal function have emerged as novel strategies to delay AD progression [68].

Microglia have been demonstrated to play a crucial role in iron metabolism within AD, not only participating in iron storage and regulation but also being closely implicated in neuroinflammation and neurodegenerative processes. These findings offer novel therapeutic targets for AD, such as inhibiting neuroinflammation and delaying disease progression by modulating iron metabolism in microglia.

### 6.2. Microglia and Energy Metabolism Reprogramming

In recent years, substantial evidence has demonstrated that abnormal cerebral glucose metabolism constitutes a hallmark pathological feature in AD patients, leading to the designation of AD as ‘type 3 diabetes’ [72]. Disrupted glucose metabolism within the brain not only compromises neuronal energy supply but also profoundly remodels the immune functional state of microglia, establishing itself as a frontier research focus in elucidating the pathogenesis of AD.

Studies have revealed that metabolic reprogramming underlies the inflammatory response of Aβ-induced microglia. In the 5xFAD mouse model, Aβ-tolerant microglia exhibit pronounced metabolic dysfunction [73]. Researchers confirmed that direct modulation of cellular metabolism exerts bidirectional regulation on microglial phenotypic changes: inhibiting the mTOR pathway and glycolysis significantly reduces Aβ-induced microglial inflammatory responses, whereas promoting glycolytic metabolism with IFN-γ restores microglial motility and enhances their phagocytic activity towards Aβ [74]. This discovery reveals the intimate relationship between cellular metabolic pathways and microglial functional phenotypes, providing a theoretical basis for targeted metabolic interventions. Under AD pathological conditions, microglial metabolism shifts from oxidative phosphorylation to aerobic glycolysis [75]. This metabolic shift leads to intracellular lactate accumulation, which is transported extracellularly via monocarboxylate transporters. Increased lactate levels in the brain induce histone H4K12 and H3K18 lactylation in microglia, which in turn activates the NF-κB signaling pathway and ultimately upregulates the expression of the pro-inflammatory cytokines IL-6 and TNF-α, thereby promoting the progression of neuroinflammation [76]. Further investigations reveal that glycolytically derived lactate can be transported into the nucleus, mediating histone lactylation—a novel epigenetic regulatory mechanism. H4K12la enhances transcriptional activity by binding to the promoter regions of glycolytic genes (such as Pkm and Ldha), thereby forming a ‘glycolysis-H4K12la-PKM2’ positive feedback loop that exacerbates glucose metabolism disorders and induces microglial dysfunction. Intervention in this circuit—such as through pharmacological inhibition of PKM2 or specific knockout of Pkm2 in microglia—significantly reduces Aβ burden, suppresses microglial activation, and improves memory and spatial learning abilities in mice [77].

Hexokinase 2 (HK2), as the first rate-limiting enzyme in glycolysis, performs a foundational role in the metabolic reprogramming of microglia [78]. Research published by the Xiamen University Medical School team in Nature Metabolism reveals HK2 induces glucose-lipid metabolic reprogramming in microglia, regulating ATP production, phagocytic function, and inflammatory responses [79]. The specific knockout of the HK2 gene in microglia, or the pharmacological inhibition of HK2, has been shown to enhance phagocytic function, increase Aβ clearance capacity, and improve cognitive function in AD mice [80]. Mechanistic studies indicate that partial inhibition of HK2 exerts protective effects by increasing levels of its cytoplasmic target IKBα, thereby suppressing NF-κB nuclear translocation and downstream inflammatory gene expression. This finding suggests that ‘moderate inhibition’ rather than ‘complete blockade’ of HK2 represents the optimal therapeutic strategy for AD. Recent research indicates that the mitochondrial dynamics-associated protein OPA1 downregulates HK2 expression, thereby promoting ATP production and Aβ clearance in microglia and improving cognitive function [81]. This provides a novel intervention approach targeting the mitochondrial-glycolytic axis.

TREM2-mediated phagocytosis by microglia is an energy-intensive process. TREM2 regulates energy metabolism reprogramming in microglia via the PI3K-AKT-mTOR-HIF-1α signaling pathway, thereby influencing phagocytic function. Research indicates that TREM2 deficiency leads to impaired metabolic adaptability in microglia, compromised mTOR signaling, and dysfunction of autophagy-lysosomal pathways, subsequently affecting energy metabolism around Aβ plaques [82].

Regulation of microglia bioenergetics pathways has emerged as a key therapeutic target in AD. A more profound comprehension of microglia metabolic characteristics across various disease stages, as well as their molecular regulatory networks, holds significant potential in the development of precision interventions that target specific metabolic pathways. These interventions may include HK2 partial inhibitors, PKM2 modulators, and AMPK agonists, with the aim of delivering novel therapeutic strategies against AD and other neurodegenerative disorders.

### 6.3. Microglia and Abnormal Lipid Metabolism

Lipid metabolism not only plays a central role in maintaining the integrity and function of cell membranes, but also profoundly participates in pathophysiological processes such as inflammatory responses, cellular signal transduction, and neuroprotection and injury. In the context of AD, the lipid metabolic pathways of microglia undergo significant reprogramming, leading to the accumulation of harmful lipids that subsequently impair neuronal function and survival.

APOE, as the primary lipid carrier protein in the central nervous system, plays a critical role in this process. The APOE4 allele is widely considered to be the strongest genetic risk factor for late-onset AD. It induces cell-type-specific lipid metabolism disorders within the brain, particularly triggering energy metabolism defects in microglia and promoting the formation of lipid droplet-accumulating microglia, thereby initiating a cascade of neurodegenerative responses [83,84]. Research indicates that APOE aggregates within the endolysosomal system of microglia can induce Aβ fibrillation and accelerate plaque deposition [66]. The role of APOE aggregation in AD extends beyond being a concomitant phenomenon of pathological deposition; it also functions as a ‘seed’ for Aβ plaque formation. Recent studies have elucidated the molecular mechanisms by which APOE4 drives lipid metabolism disorders in microglia. APOE4 induces lipid metabolism imbalance through PU.1/NF-κB-mediated transcriptional reprogramming and activation of the endoplasmic reticulum stress-SREBP2 pathway. These dysregulations have been shown to exacerbate neuroinflammatory responses, promote lipid droplet accumulation and cholesterol overload, impair lysosomal function, and ultimately diminish the phagocytic capacity of microglia. Mechanistically, APOE4 has also been demonstrated to activate neuroinflammation via the LilrB3-mediated type I interferon signaling pathway. Tony Wyss-Coray’s team at Stanford University School of Medicine used single-cell RNA sequencing to analyze brain tissue from AD patients. This analysis revealed a microglial state defined by the expression of the enzyme ACSL1, which is associated with lipid droplets [85]. Research indicates that ACSL1-positive microglia are most abundant in APOE4/4 genotype AD patients, suggesting a link between the APOE4/4 genotype and significant lipid droplet accumulation in AD microglia. These LDAM have been shown to promote tau phosphorylation and neurotoxicity in an APOE-dependent manner [85]. Further investigations demonstrate that LDAM exhibit a pro-inflammatory, hypophagocytic phenotype [86]. Furthermore, the APOE4-induced lipid accumulation state has been demonstrated to diminish microglial responsiveness to neuronal activity signals [87]. The findings indicate that the APOE4 genotype promotes the transformation of microglia into an evolutionarily conserved, maladaptive and destructive LDAM state in response to innate immune triggers, including Aβ [85], offering novel insights into the pathological mechanisms of AD.

Microglia lipid metabolism dysfunction occupies a central position in the pathology of AD. A novel therapeutic direction for AD has emerged, focusing on targeting APOE4-related pathways. This involves the implementation of strategies such as modulating lipid metabolism, intervening in lipid droplet formation, and restoring lysosomal function.

Metabolically, microglial phenotype is fundamentally shaped by bioenergetic status. Iron accumulation impairs Aβ phagocytosis via complement C3 upregulation and APOE-TREM2 axis suppression; glycolytic reprogramming via HK2 and PKM2 drives pro-inflammatory lactate production and histone lactylation; and APOE4-driven LDAM critically compromises phagocytic capacity and neuronal surveillance. These metabolic alterations are not merely consequences of activation but active determinants of functions. Table 3 integrates the major metabolic pathways underlying microglial functional transitions in AD, encompassing iron, glucose, and lipid metabolism, as well as emerging epigenetic (histone lactylation) and mitochondrial regulatory mechanisms.

## 7. Role of Microglia–Astrocyte Interactions in the Progression of AD

Microglia and astrocytes, as the two principal glial cell types within the central nervous system, exhibit intricate interactions during the pathological progression of AD. Recent studies have revealed that they form functional networks through multiple molecular signaling pathways, cooperatively regulating neuroinflammation, Aβ clearance, tau pathology propagation, and synaptic function. Imbalances in this intercellular communication may represent a key driver of AD progression.

### 7.1. Molecular Signaling Networks in Glial Cell Interactions

#### 7.1.1. IL-3/IL-3Rα Signaling Axis

IL-3, as a key regulatory molecule derived from astrocytes, plays a central role in modulating microglial function. Research indicates that astrocytes constitute the primary source of IL-3, whilst microglia highly express the IL-3 receptor IL-3Rα. Under pathological conditions of AD, the absence of IL-3 signaling can cause microglia to transition from an activated state to a quiescent state, thereby diminishing their capacity to migrate towards and clear Aβ plaques. Exogenous supplementation of IL-3 significantly reduces Aβ plaque burden and improves cognitive function in mouse brains [88]. This discovery reveals the molecular mechanism by which astrocytes actively regulate microglial function via IL-3 signaling, offering new insights into glial cell interactions.

#### 7.1.2. Complement C3/C3aR Signaling Pathway

The complement system performs a dual function in glial cell interactions. C3 is primarily secreted by astrocytes, with its receptor C3aR expressed on the surface of microglia. Activation of the C3/C3aR pathway exerts time-dependent effects on the Aβ clearance function of microglia: acute activation enhances phagocytic capacity, whereas chronic sustained activation leads to functional exhaustion and clearance impairment. The pharmacological inhibition of C3aR has been shown to simultaneously alleviate Aβ and tau pathology, restore synaptic function, and correct immunodysregulation [89]. This finding emphasizes the necessity for precise regulation of complement signaling in therapeutic interventions for AD.

#### 7.1.3. Rack1-IGF1 Signaling Axis

Recent research has revealed the pivotal role of the scaffold protein Rack1 in regulating communication between microglia and astrocytes. The present study has demonstrated that in both AD patients and mouse models, microglia exhibit significantly elevated levels of Rack1. The microglial-specific knockout of Rack1 has been demonstrated to reduce Aβ deposition, alleviate neuroinflammation, and enhance cognitive function. Mechanistic studies indicate that Rack1 deficiency reduces microglial numbers and diminishes their ability to engulf Aβ. Conversely, the observed outcomes suggest a promotion of astrocyte proliferation and an enhancement of their phagocytic activity, a phenomenon that is concomitant with an upregulation of insulin-like growth factor 1 (IGF1) levels [90]. Inhibition of the IGF1 receptor (IGF1R) blocked this protective effect, confirming the central role of the Rack1-IGF1-IGF1R signaling axis in mediating glial functional redistribution. This finding unveils, for the first time, a mechanism by which microglia can indirectly compensate for their own functional deficits by regulating astrocytic phagocytic function, thus providing a novel therapeutic target for the treatment of AD.

#### 7.1.4. C1q-Dependent Synaptic Clearance Mechanism

C1q, as the initiating molecule of the classical complement pathway, serves a critical role in mediating synaptic clearance by glial cells. In the TauP301S mouse model, C1q deficiency significantly reduces synaptic phagocytosis by astrocytes and microglia, thereby restoring synaptic density. It is noteworthy that these two glial cell types exhibit functional specialization in terms of synaptic clearance: astrocytic lysosomes enrich excitatory synaptic markers, whereas microglial lysosomes contain greater quantities of inhibitory synaptic substances. In AD models exhibiting concurrent Aβ and tau pathology, TREM2 deficiency impaired synaptic phagocytosis by microglia, while astrocytes compensatorily enhanced clearance of inhibitory synapses surrounding plaques. This finding reveals synergistic and compensatory mechanisms within glial cells for maintaining synaptic homeostasis.

#### 7.1.5. AHNAK: Key Driver of Disrupted Glia-Neuron Communication

Recent proteomics studies have revealed through unsupervised network analysis that the AHNAK protein serves as a central driver of disrupted communication between neurons and glial cells. AHNAK is primarily expressed in astrocytes, with its levels significantly elevated in the brains of AD patients and positively correlated with Aβ and tau protein burdens. In a human pluripotent stem cell-derived brain cell co-culture model, reducing AHNAK expression decreased tau protein levels and improved neuronal function [91]. This discovery provides a novel entry point for understanding the molecular mechanisms underlying the breakdown of glia-neuron communication.

### 7.2. Functional Effects of Glial Cell Interactions

#### 7.2.1. Synergistic Amplification of Neuroinflammation

The formation of a positive feedback amplification loop between microglia and astrocytes has been identified as a key feature of AD neuroinflammation. The release of IL-1, TNF-α, and C1q by activated microglia induces astrocytes to transition towards a neurotoxic A1 phenotype [92,93]. Conversely, A1 astrocytes secrete pro-inflammatory factors that further activate microglia. This mutual activation perpetually amplifies the inflammatory cascade, exacerbating neuronal damage. Spatiotemporal transcriptomics studies further reveal that the accumulation of microglia around Aβ plaques disrupts astrocytic signaling, leading to an imbalance in GABAergic/glutamatergic signaling within hippocampal neurons and triggering synaptic dysfunction.

#### 7.2.2. Synergistic Regulation of Aβ Clearance

Glial cells exhibit a dual relationship of synergy and compensation in Aβ clearance. Under physiological conditions, microglia serve as the primary Aβ clearance cells, while astrocytes enhance microglial function by secreting factors such as TGF-β [94]. When microglial function is impaired (e.g., TREM2 deficiency or Rack1 knockout), astrocytes can compensate by upregulating phagocytic activity [90]. Recent research has revealed the pivotal role of astrocytic ‘immune priming’ in regulating microglial Aβ clearance. Transient immune stimulation induces a primed state in astrocytes; upon subsequent Aβ exposure, these primed astrocytes secrete cytokines that promote microglial Aβ uptake. Recent research has revealed the pivotal role of astrocytic ‘immune priming’ in regulating microglial Aβ clearance. The induction of a primed state in astrocytes by transient immune stimulation has been demonstrated; subsequent exposure to Aβ has been shown to result in the secretion of cytokines by these primed astrocytes, which in turn promote microglial uptake of Aβ. However, astrocytes that carry the APOE4 risk allele exhibit impaired priming capacity, resulting in ineffective support of microglial phagocytosis. This finding provides novel insights into the mechanisms underlying APOE4’s increased risk for AD [94].

#### 7.2.3. Synergistic Propagation of Tau Pathology

Glial cell interactions also participate in regulating tau pathology. Research indicates that IL-3 secreted by astrocytes modulates the tau clearance capacity of microglia. C3aR signaling not only influences Aβ clearance but also directly regulates tau pathology and synaptic integrity.

Targeting the microglia–astrocyte interaction pathway has emerged as a novel therapeutic strategy for AD. Current research directions include: IL-3/IL-3Rα agonists promoting Aβ clearance; C3aR antagonists simultaneously ameliorating Aβ and tau pathology; Rack1 inhibitors enhancing astrocytic compensatory function via IGF1 signaling; AHNAK neutralizing antibodies restoring neuron-glia communication; and screening for small-molecule modulators based on intercellular communication mechanisms.

Intercellularly, microglia–astrocyte crosstalk through IL-3, C3/C3aR, and Rack1-IGF1 signaling creates synergistic networks for both Aβ clearance and neuroinflammation amplification. Astrocytic “immune priming” can enhance microglial phagocytosis, though this capacity is impaired by the APOE4 genotype, providing a mechanistic link between genetic risk and glial dysfunction.

## 8. Current Limitations and Future Directions

Species and Model Limitations: The majority of current findings derive from transgenic mouse models (e.g., 5XFAD, APP/PS1, PS19) that imperfectly recapitulate human AD pathology, particularly regarding neuroinflammation dynamics and aging. Recent single-cell studies have revealed substantial transcriptomic divergence between murine and human microglia, suggesting that some DAM and TIM signatures may not be fully conserved across species. Furthermore, most models rely on familial AD mutations, whereas sporadic AD—which constitutes >95% of cases—involves distinct risk factors such as APOE4 and TREM2 variants that modify microglial responses differently.

Temporal and Spatial Resolution: The precise temporal sequence of microglial state transitions in human AD brains remains poorly defined. Current cross-sectional studies cannot capture the dynamic trajectory from homeostatic surveillance to DAM activation, functional exhaustion, and ultimately death or senescence. Additionally, microglia exhibit marked regional heterogeneity (e.g., hippocampus vs. cortex vs. white matter), yet most studies aggregate these populations, obscuring region-specific therapeutic vulnerabilities.

Technical Constraints: Existing in vitro systems—including two-dimensional cultures and cerebral organoids—lack the full complexity of the brain’s immune environment, particularly meningeal, perivascular, and choroid plexus immune compartments that contribute to AD neuroinflammation. Moreover, current microglial depletion-repopulation strategies (e.g., CSF1R inhibitors) lack temporal precision and may cause off-target effects on other macrophage populations.

## 9. Future Research Directions

Multi-omics integration and dynamic atlases: Future studies should combine single-cell transcriptomics, spatial transcriptomics, proteomics, and metabolomics to construct four-dimensional atlases of microglial states across AD progression. Integrating these datasets with advanced machine learning algorithms may identify previously unrecognized microglial substates and their transition trajectories, enabling the definition of precise therapeutic windows.

Precision microglial modulation: Rather than broad immunosuppression or activation, the next generation of therapeutics should target specific microglial states. Promising directions include: (1) CAR-microglia engineered for enhanced Aβ/tau clearance; (2) TIM-3 immune checkpoint modulators to reverse functional exhaustion; (3) metabolic reprogramming agents (HK2 partial inhibitors, iron chelators, APOE4-targeted lipid modulators) to restore bioenergetic homeostasis; and (4) mechanobiological interventions targeting Piezo1 to enhance plaque compaction.

In vivo microglial imaging and biomarker development: Advancing TSPO-PET, tau-PET, and cerebrospinal fluid sTREM2 monitoring will enable real-time tracking of microglial dynamics in living patients. Coupling these biomarkers with peripheral immune profiling (e.g., clonal CD8^+^ T cell expansion in CSF) could facilitate personalized immunotherapy, allowing clinicians to tailor microglial-modulating treatments to individual pathological stages and immune profiles.

## 10. In Summary

Microglia exert multifaceted roles in the pathogenesis and progression of AD, including promoting neuroinflammation, participating in Aβ and tau clearance and propagation, influencing iron metabolism, and interacting with astrocytes. Modulating microglial function offers unique therapeutic advantages, potentially halting pathological progression at early stages. Targeting specific genes or signaling pathways can reduce pro-inflammatory cytokine production, inhibit the transformation into DAM, and prevent neuronal damage. Concurrently, inducing microglia to repair neural tissue, phagocytose debris and plaques, and maintain normal metabolic processes can prevent disease triggered by abnormal activation.

From a therapeutic standpoint, we posit that the future of AD treatment lies not in broadly suppressing microglial activity—which risks compromising essential immune surveillance—but in precisely calibrating their functional states according to disease stage. Early-stage intervention (preclinical to MCI) should prioritize enhancing TREM2/SYK-mediated phagocytosis and promoting M2/DAM polarization through metabolic reprogramming and astrocytic priming. In contrast, advanced-stage strategies must address TIM exhaustion and iron/lipid-mediated metabolic dysfunction via immune checkpoint modulation (e.g., TIM-3), iron chelation, and lysosomal restoration. The integration of single-cell technologies with metabolic profiling and in vivo imaging will be instrumental in identifying patient-specific microglial signatures, ultimately paving the way for personalized immunometabolic therapies that can halt—or potentially reverse—AD pathological progression.

## Figures and Tables

**Table 1:** Key Signaling Pathways in Microglial Aβ Metabolism.

Pathway	Primary Function	Effect on Aβ	Therapeutic Target
REM2/SYK	Phagocytosis & plaque compaction	Promotes clearance & barrier formation	TREM2 agonists, SYK activators
Piezo1	Mechanotransduction & Aβ sensing	Enhances uptake via stiffness sensing	Piezo1-targeted nanobubbles
TAM	Anti-inflammatory Aβ clearance	Trims plaques, limits tau spread	Gas6 fusion proteins
PTP1B-SYK	Negative regulation of phagocytosis	Inhibition promotes clearance	PTP1B inhibitors
Clec7a-SYK	Synaptic loss in tauopathy	Modulates synaptic pruning	Anti-CLEC7A nanobodies
TMEM119-LRP1	Lysosomal Aβ degradation	Direct binding and degradation	TMEM119 enhancers

**Table 2:** Microglial Phenotypes and Their Roles in Tau Pathology.

Phenotype	Key Markers	Role in Tau Pathology	Therapeutic Implication
Homeostatic	P2RY12, CX3CR1, TMEM119	Baseline surveillance	Preserve homeostatic state
DAM	TREM2, Cst7, Lpl, Tyrobp	Phagocytic clearance of tau seeds	Enhance TREM2/SYK signaling
TIM	HAVCR2 (TIM-3), APOE, C1q	Functional exhaustion	TIM-3 modulators (ATRA)
Rod-like	IFN-γ response genes	Correlates with neurodegeneration	Interferon pathway inhibitors
M1	CD86, iNOS, TNF-α, IL-6	Promotes tau propagation	NLRP3 inhibitors, Gal-9 blockade
M2	CD206, Arg1, IL-10, TGF-β	Protective clearance	Promote M2 polarization

**Table 3:** Metabolic Reprogramming in AD Microglia.

Metabolic Pathway	Key Regulators	AD-Associated Change	Therapeutic Strategy
Iron Metabolism	DMT1, Ferritin, GPX4, TRPM8	Iron overload → ferroptosis	Iron chelators; GPX4 activators; CIS therapy
Glycolysis (HK2/PKM2)	HK2, PKM2, mTOR, HIF-1α	Shift to aerobic glycolysis	HK2 partial inhibitors; PKM2 modulators
Lipid Metabolism (APOE4/LDAM)	APOE4, ACSL1, SREBP2	Lipid droplet accumulation	APOE4-targeted lipid modulators
Oxidative Phosphorylation	TREM2-PI3K-AKT-mTOR axis	Impaired in TREM2 deficiency	TREM2 agonists
Histone Lactylation (H4K12la)	Lactate, MCTs, NF-κB	Glycolysis-H4K12la-PKM2 loop	PKM2 inhibitors
Mitochondrial Dynamics (OPA1)	OPA1, mitochondrial fusion	Enhanced ATP production	OPA1 enhancers

## Data Availability

No new data were created or analyzed in this study. Data sharing is not applicable to this article.
